# Relationships between age at first substance use and persistence of cannabis use and cannabis use disorder

**DOI:** 10.1186/s12889-021-11023-0

**Published:** 2021-05-27

**Authors:** Seán R. Millar, Deirdre Mongan, Bobby P. Smyth, Ivan J. Perry, Brian Galvin

**Affiliations:** 1 Health Research Board, Grattan House 67–72 Lower Mount Street, Dublin, Ireland; 2 School of Public Health, University College Cork, 4th Floor, Western Gateway Building, Cork, Ireland; 3 Department of Public Health & Primary Care, Trinity College Dublin, Russell Building, Tallaght Cross, Dublin, Ireland

**Keywords:** Cannabis, Use, Abuse, Dependence, Age, Onset, Tobacco, Alcohol

## Abstract

**Background:**

From a secondary prevention perspective, it is useful to know who is at greatest risk of progressing from substance initiation to riskier patterns of future use. Therefore, the aim of this study was to determine relationships between age at first use of alcohol, tobacco and cannabis and patterns of cannabis use, frequency of use and whether age of substance use onset is related to having a cannabis use disorder (CUD).

**Methods:**

We analysed data from Ireland’s 2010/11 and 2014/15 National Drug Prevalence Surveys, which recruited 5134 and 7005 individuals respectively, aged 15 years and over, living in private households. We included only those people who reported lifetime cannabis use. Multinomial, linear and binary logistic regression analyses were used to determine relationships between age of substance use onset and patterns of cannabis use, frequency of use and having a CUD.

**Results:**

When compared to former users, the odds of being a current cannabis user were found to be reduced by 11% (OR = 0.89; 95% CI: 0.83, 0.95) and 4% (OR = 0.96; 95% CI: 0.92, 1.00) for each year of delayed alcohol and cannabis use onset, respectively. Among current users, significant inverse linear relationships were noted, with increasing age of first use of tobacco (β = − 0.547; *P* < .001) and cannabis (β = − 0.634; *P* < .001) being associated with a decreased frequency of cannabis use within the last 30 days. The odds of having a CUD were found to be reduced by 14% (OR = 0.86; 95% CI: 0.78, 0.94) and 11% (OR = 0.89; 95% CI: 0.82, 0.98) for each year of delayed tobacco and cannabis use onset respectively in analyses which examined survey participants aged 15–34 years.

**Conclusions:**

Among people who report past cannabis use, it is those with a more precocious pattern of early use of substances, including alcohol, and especially tobacco and cannabis, who are more likely to report ongoing, heavy and problematic cannabis use. Secondary prevention initiatives should prioritise people with a pattern of very early onset substance use.

## Introduction

Cannabis is the third most commonly used recreational drug in the world (after alcohol and nicotine) [1]. In the Republic of Ireland, the most recent national survey, conducted in 2014/15, found that 27.9% of people aged 15–64 years had used cannabis at some point in their lives, with 7.7% and 4.4% having used cannabis within the last year or last month respectively [2]. These prevalence figures are considerably higher than those recorded for any other illegal drug and have increased considerably since 2002 [3, 4].

Cannabis use disorder (CUD) is now the most common disorder present in new addiction treatment episodes in the European Union [5]. Across Europe, the number of first-time treatment entrants for primary cannabis use has continuously increased from around 43,000 (28% of all new entrants into drug addiction treatment) in 2006 to around 75,000 (47% of all new treatment entrants) in 2015 [6]. In Ireland, coinciding with an increase in cannabis use, there has also been a substantial increase in treated cannabis addiction and cannabis-related psychiatric problems [7]; among first-time treatment entrants, cannabis is the most frequently reported main problem drug, accounting for 38% of all new entrants [8].

There is ongoing debate regarding relationships between early onset substance use and later use of other drugs [9]. The ‘common liability model’ states that a combination of risk factors places some people at increased risk of both early initiation and of subsequent progression to more serious and sustained drug abuse [10]. Biological, genetic and environmental factors may contribute to risk of early onset of substance use [11]. Neurobiological research has suggested that alcohol, tobacco and other drugs seem to apply to the same neurotransmitters in the brain, proposing a chain of causation between the use of alcohol and cigarettes and subsequent use of cannabis and other drugs [12]. During adolescence there is substantial remodelling of the brain and the endocannabinoid system plays a key role in this process [11, 13]. Animal and human studies indicate that the brain may be particularly vulnerable to the effects of alcohol, nicotine and cannabis during these adolescent years while the remodelling process is ongoing [11, 14–16]. Lubman et al. have discussed a ‘two-hit’ model, where individuals who have a combination of biological and environmental risk factors are more likely to use substances during adolescence, and that substance use then causes a neurobiological alteration which further accelerates progression to a substance use disorder [11]. There is also evidence that young people respond differently to cannabis compared to older adults, showing reduced satiety, and this may lead them to use cannabis more heavily [17]. Recent research from the United States has shown that adolescent onset users of cannabis are more likely to develop a CUD than young adults [18]. It has also been proposed that the link between earlier use of substances and later heavier cannabis use and CUD is symptomatic of people who are at risk of psychosocial disorders [19].

Although numerous studies have examined relationships between early onset drinking, tobacco and cannabis use with later drug use [9, 20], this research has tended to focus on a narrow 12–25-year age range. In addition, fewer studies have explored factors associated with progression to ongoing, heavier and problematic cannabis among lifetime cannabis users. It is also unclear whether associations between younger age at substance use onset and cannabis use patterns are independent of other influential factors that may constitute an underlying vulnerability for heavier substance use and substance use disorders [20].

Further research on relationships between age of substance use onset and cannabis is needed. Such knowledge is important to guide future regulation systems, to inform both clinical and public health practice and for assessing drug policy. This is particularly relevant in Ireland at the present time given the increase in number of people being treated for cannabis addiction as well as calls for liberalising cannabis laws [7, 21]. Therefore, the aim of this study was to determine associations between age at first use of alcohol, tobacco and cannabis and cannabis use patterns, drawing on data from two large nationally representative studies which used the same field survey procedures and data collection methods. In particular, we explored how age at first substance use relates to frequency of cannabis use among current users and whether individuals with a CUD are more likely to be earlier users of alcohol, tobacco or cannabis.

## Methods

### Study population and setting

We analysed data from Ireland’s 2010/11 and 2014/15 Drug Prevalence Surveys. These national, cross-sectional studies recruited stratified clustered samples of individuals aged 15 years and over living in private households in Ireland. The numbers interviewed in 2010/11 and 2014/15 were 5134 and 7005, respectively. For both surveys, the sampling frame used was the An Post/Ordnance Survey Ireland GeoDirectory database, which is a list of all addresses in the Republic of Ireland and distinguishes between residential and commercial establishments. A three-stage process was used to construct the sample for each survey. The first stage involved stratifying the population into 10 former health board regions in Ireland. In the second stage of stratification, electoral divisions were selected as the primary sampling units across the 10 former health board regions. Before selection, the primary sampling units were ranked by the following sociodemographic indicators: population density, male unemployment and social class, according to the year of data collection, to ensure that a representative cross-section of areas were included. Finally, in each primary sampling unit, addresses were chosen randomly, and at each address, one person was selected to participate in the survey using the ‘last birthday’ rule. For each survey, the achieved sample was weighted by sex, age and former health board region to maximise its representativeness of the general population. The surveys involved both pre-weights and post-stratification (PS) weights. Pre-weights were used to allow for different selection probabilities of each end respondent, while the PS weights were used to scale up the responses within each demographic cell to represent their respective target populations. A more comprehensive description of the methodology used in the surveys has been detailed elsewhere [22, 23].

The surveys involved face-to-face interviews in the home of each participant and a self-completed questionnaire. The home interviews were conducted by trained interviewers using Computer Assisted Personal Interviewing (CAPI). Fieldwork for the surveys was carried out between October 2010 to May 2011 and August 2014 to August 2015 and both achieved a 60% response rate. All data collection methods were performed in accordance with relevant guidelines and regulations. No data on non-respondents were collected. The studies were granted ethical approval by the Research Ethics Committee of the Royal College of Physicians in Ireland and signed informed consent was obtained from the parental guardians of minors and from subjects 18+ years of age for data to be used for research purposes. Ireland’s National Drug Prevalence Surveys are GDPR compliant [4].

### Measures

#### Outcome variables

##### Cannabis use

Patterns of cannabis use were defined via the European Monitoring Centre for Drugs and Drug Addiction (EMCDDA) criteria [24]. Respondents who had used cannabis at some point in their lives were classified into one of three mutually exclusive groups. These groups were former users (used cannabis at least once in their life but not in the past year), recent users (used cannabis at least once within the past year but not in the past month) or current users (used cannabis at least once within the past month). Frequency of cannabis use among current users was determined by asking participants: “During the last 30 days on how many days have you taken cannabis?” This was coded as 1–30 days. Data on frequency of use were available for 394 respondents.

##### Cannabis use disorder (CUD)

The Diagnostic and Statistical Manual of Psychiatric Disorders (DSM), better known as the DSM-IV, is published by the American Psychiatric Association and covers all mental health disorders for children and adults. Substance abuse and dependence is defined by the DSM-IV as a maladaptive pattern of substance use leading to clinically significant impairment or distress [25, 26]. In this study, cannabis abuse and cannabis dependence were classified according to DSM-IV criteria and were measured via a self-completed questionnaire using the four items that denote cannabis abuse and seven items that denote cannabis dependence from the Composite International Diagnostic Interview (CIDI). Cannabis abuse was established from a positive response in one or more of the four domains on the DSM-IV diagnostic criteria in the 12 months before the interview. Cannabis dependence was determined from a positive response in three or more of the seven domains on the DSM-IV in the 12 months before the interview. CUD was determined as any cannabis abuse or dependence in the 12 months prior to the interview.

The CIDI is a widely accepted and frequently used operationalisation of the DSM-IV. Advised by the EMCDDA, the abbreviated version, the Munich Composite International Diagnostic Interview (M-CIDI), a 19-item instrument reflecting the four cannabis abuse and the seven cannabis dependence criteria, was used for both the 2010/11 and 2014/15 Drug Prevalence Surveys [27].

#### Exposure variables

Age at first use of alcohol, tobacco and cannabis was obtained from questions which asked “At which age did you first try substance x?” Data on age of first substance use were available for 2901 (alcohol), 2371 (tobacco) and 2956 (cannabis) subjects. Independent variables likely associated with alcohol, tobacco and cannabis use were identified through the literature [4]. Demographic variables selected were sex, age and marital status. Additionally, geographic region (Dublin/outside Dublin) was included to control for any possible effects of sociopolitical and cultural factors present within Ireland. Educational attainment, employment, housing tenure, other lifetime drug use (use of cocaine, ecstasy, amphetamines or sedatives) and current use of alcohol or tobacco were also included as covariates in analyses.

### Statistical analysis

The datasets were codified, affixed with a timestamp variable (year) and merged. Study participants who had never used cannabis in their lives were excluded from analyses. Descriptive characteristics were examined according to cannabis use. Displayed frequencies and percentages are weighted. Multinomial logistic regression analyses were used to examine associations between age at first use of alcohol, tobacco and cannabis (as linear variables) and cannabis use patterns. Linear regression was used to test associations between age at first substance use and frequency of cannabis use among subjects who had used cannabis within the last month. Binary logistic regression was used to explore relationships between age at first substance use and CUD (defined as either cannabis abuse or dependence) among subjects who reported using cannabis in the last year. For all regression analyses, an unadjusted model was used to test associations and a second model was adjusted for sex and age. A third model was adjusted for sex, age, marital status, region, education, employment, housing tenure, other lifetime drug use, current alcohol or tobacco use and year of data collection. To examine age-related results, we also compared full sample models to age stratified models in multinomial logistic regression analyses; young adults (aged 15–34 years) vs. older adults (aged 35+ years). These classifications are recommended by the EMCDDA for use in general population surveys [24]. Due to sample size constraints, models which examined age at first substance use with frequency of cannabis use or CUD compared all subjects aged 15 years or over to young adults. There were low levels of missing data. Missing independent variable data were thus assumed to be ignorable and missing at random.

Data analysis was conducted using Stata SE Version 15.1 (Stata Corporation, College Station, TX, USA) for Windows. For all analyses, a *P*-value (two-tailed) of less than .05 was considered to indicate statistical significance.

## Results

### Descriptive characteristics

Among both surveys there were 2979 (weighted prevalence = 24.6%) subjects who indicated having used cannabis at some point in their lives. Characteristics of these subjects according to former, recent and current use, and CUD, are shown in Table 1. The weighted prevalence of former cannabis use was 74.4% for the combined surveys, while the weighted prevalence of recent and current cannabis use was 12.1% and 13.5% of participants, respectively. Among subjects who reported former cannabis use, 53.4% were aged 35 years or older; 76.8% and 77.7% of participants who indicated recent or current use were between 15 and 34 years of age. A higher percentage of survey participants who reported former, recent or current cannabis use were also male. Within the subset of people who reported recent and current use, 33.1% reported a CUD defined as either cannabis abuse or dependence. Compared to people who reported former use, participants who indicated recent or current cannabis use, or who had a CUD, were more likely to be single or had never married, had lower educational levels, were less likely to be employed and were more likely to be living in rented accommodation or with family/friends.

**Table 1:** Characteristics of subjects who reported lifetime cannabis use – full sample and according to former, recent and current use and for those with a cannabis use disorder

Variable	Full sample ***n*** = 2979	Former cannabis use ***n*** = 2215	Recent cannabis use ***n*** = 362	Current cannabis use ***n*** = 402	Cannabis use disorder^**a**^ ***n*** = 253
**Sex (%)**					
Male	1923 (64.4)	1361 (61.4)	247 (68.2)	316 (78.4)	207 (81.8)
**Age group (%)**					
35+	1354 (45.5)	1180 (53.4)	84 (23.2)	90 (22.4)	38 (15.0)
25–34	1102 (37.1)	816 (36.9)	128 (35.4)	159 (39.6)	89 (35.2)
15–24	518 (17.4)	215 (9.7)	150 (41.4)	153 (38.1)	126 (49.8)
**Marital status (%)**					
Married/cohabiting	1642 (55.2)	1409 (63.7)	108 (29.9)	125 (31.0)	64 (25.3)
Divorced/separated/widowed	156 (5.2)	125 (5.7)	11 (3.0)	20 (5.0)	8 (3.2)
Single/never married	1178 (39.6)	678 (30.7)	242 (67.0)	258 (64.0)	181 (71.5)
**Region (%)**					
Dublin (compared to outside Dublin)	1133 (38.0)	819 (37.0)	134 (37.0)	180 (44.7)	101 (39.9)
**Education (%)**					
Completed third level	1677 (56.4)	1327 (60.0)	179 (49.6)	171 (42.6)	86 (34.3)
Completed secondary	998 (33.5)	699 (31.6)	146 (40.4)	152 (37.9)	99 (39.4)
Primary/none	299 (10.1)	185 (8.4)	36 (10.0)	78 (19.5)	66 (26.3)
**Employment (%)**					
Employed	1875 (63.0)	1517 (68.5)	179 (49.4)	180 (44.7)	101 (39.9)
Unemployed	527 (17.7)	341 (15.4)	73 (20.2)	113 (28.0)	81 (32.0)
Student	321 (10.8)	141 (6.4)	97 (26.8)	83 (20.6)	57 (22.5)
Other	256 (8.6)	215 (9.7)	13 (3.6)	27 (6.7)	14 (5.5)
**Housing tenure (%)**					
Owned	1657 (55.6)	1379 (62.3)	165 (45.5)	113 (28.1)	75 (29.6)
Rented	1033 (34.7)	687 (31.0)	144 (39.7)	203 (50.5)	118 (46.6)
Living with family/friends or other	289 (9.7)	149 (6.7)	54 (14.9)	86 (21.4)	60 (23.7)
**Other lifetime drug use (%)**					
Yes	1410 (47.3)	938 (42.3)	181 (50.0)	291 (72.2)	187 (73.9)
**Current alcohol use (%)**					
Yes	2452 (82.4)	1781 (80.4)	324 (89.5)	348 (86.6)	211 (83.1)
**Current tobacco use (%)**					
Yes	1417 (47.6)	867 (39.2)	226 (62.4)	324 (80.6)	197 (77.9)
**Age at first use (mean)**^**b**^					
***All subjects***					
Alcohol	15.98 ± 2.2	16.19 ± 2.3	15.59 ± 1.7	15.20 ± 1.8	15.15 ± 1.7
Tobacco	15.50 ± 3.3	15.62 ± 3.4	15.55 ± 2.8	14.94 ± 3.1	14.75 ± 2.7
Cannabis	19.16 ± 5.0	19.66 ± 5.2	18.14 ± 4.0	17.27 ± 3.6	16.84 ± 3.4
***Subjects aged 15–34 years***					
Alcohol	15.61 ± 1.6	15.75 ± 1.6	15.47 ± 1.6	15.26 ± 1.7	15.22 ± 1.7
Tobacco	15.28 ± 2.6	15.30 ± 2.7	15.48 ± 2.6	15.09 ± 2.4	14.65 ± 2.5
Cannabis	17.74 ± 2.6	18.07 ± 2.6	17.48 ± 2.5	16.85 ± 2.4	16.52 ± 2.5
***Subjects aged 35+ years***					
Alcohol	16.44 ± 2.7	16.58 ± 2.8	15.98 ± 2.0	14.98 ± 2.2	14.78 ± 1.7
Tobacco	15.76 ± 3.9	15.89 ± 3.8	15.76 ± 3.4	14.44 ± 4.9	15.25 ± 3.5
Cannabis	20.85 ± 6.4	21.06 ± 6.4	20.28 ± 6.5	18.68 ± 6.0	18.59 ± 6.3

Displayed frequencies, percentages (in brackets) and means ± one standard deviation are weighted. Numbers may not add up in column totals because of missing data

^a^Any cannabis abuse or dependence

^b^Data on age of first substance use were available for 2901 (alcohol), 2371 (tobacco) and 2956 (cannabis) subjects

Among all subjects, 78 (2.6%) indicated having never used alcohol; 608 (20.4) had never used tobacco. Among respondents who had used cannabis in the last year, 14 (1.8%) and 101 (13.2%) had never used alcohol or tobacco respectively; for subjects who indicated having a CUD, 9 (3.6%) and 23 (9.1%) had never tried alcohol or tobacco. Mean age at first substance use was similar between the survey time periods: mean age of first use of alcohol was 16.01 in 2010/11 vs. 15.96 in 2014/15; mean age of first use of tobacco was 15.45 in 2010/11 compared to 15.55 in 2014/15; mean age of first cannabis use was 19.10 in 2010/11 vs. 19.20 in 2014/15. For the combined samples, the mean age of first use of alcohol and cannabis among younger subjects aged 15–34 years was noticeably lower than for participants aged 35 years or older. Among all subjects, mean age at first substance use was also found to be lower among people who reported recent or current use, and among individuals with a CUD, compared to survey participants who reported former cannabis use.

### Relationships between age at first substance use and cannabis use patterns

Table 2 displays odds ratios for the associations between age at first substance use and cannabis use patterns among participants who had used each substance. Among all subjects, the odds of being a current cannabis user (compared to a former user) were found to be reduced by 11% (OR = 0.89; 95% CI: 0.83, 0.95) and 4% (OR = 0.96; 95% CI: 0.92, 1.00) for each year of delayed alcohol and cannabis use onset, respectively. In stratified models, the relationship between current use and age at first use of cannabis persisted among subjects aged 15–34 years (OR = 0.93; 95% CI: 0.86, 0.99). For survey participants 35 years or older who had used cannabis at some point in their lives, the odds of being a current cannabis user were found to be reduced by 19% (OR = 0.81; 95% CI: 0.73, 0.91) for each year of delayed alcohol use onset.

**Table 2:** Associations between age at first substance use and cannabis use patterns among subjects who reported lifetime cannabis use – multinomial logistic regression (n = 2979)

Age at first use	Cannabis use patterns					
	Recent use vs. former use			Current use vs. former use		
	OR	95% CI	***P***-value	OR	95% CI	***P***-value
***All subjects***						
**Alcohol** (*n* = 2901)						
Model 1	0.86	0.81, 0.91	<.001	0.77	0.72, 0.81	<.001
Model 2	0.93	0.87, 0.99	.036	0.82	0.76, 0.87	<.001
Model 3	0.95	0.89, 1.02	.164	0.89	0.83, 0.95	.001
**Tobacco** (*n* = 2371)						
Model 1	0.99	0.96, 1.03	.757	0.93	0.90, 0.97	<.001
Model 2	1.01	0.97, 1.06	.627	0.93	0.89, 0.97	.001
Model 3	1.02	0.98, 1.07	.286	0.97	0.92, 1.02	.19
**Cannabis** (*n* = 2956)						
Model 1	0.91	0.88, 0.94	<.001	0.83	0.80, 0.86	<.001
Model 2	0.99	0.96, 1.03	.71	0.89	0.85, 0.93	<.001
Model 3	1.01	0.97, 1.04	.75	0.96	0.92, 1.00	.036
***Subjects aged 15–34 years***						
**Alcohol** (*n* = 1584)						
Model 1	0.90	0.82, 0.98	.011	0.83	0.76, 0.90	<.001
Model 2	0.96	0.88, 1.06	.434	0.87	0.80, 0.95	.002
Model 3	1.00	0.91, 1.10	.969	0.95	0.86, 1.04	.264
**Tobacco** (*n* = 1289)						
Model 1	1.03	0.97, 1.09	.377	0.97	0.92, 1.02	.251
Model 2	1.05	0.98, 1.11	.157	0.97	0.92, 1.03	.333
Model 3	1.06	0.99, 1.14	.066	0.99	0.93, 1.06	.869
**Cannabis** (*n* = 1608)						
Model 1	0.92	0.87, 0.97	.001	0.82	0.78, 0.87	<.001
Model 2	0.99	0.94, 1.06	.844	0.87	0.82, 0.92	<.001
Model 3	1.01	0.95, 1.08	.768	0.93	0.86, 0.99	.027
***Subjects aged 35+ years***						
**Alcohol** (*n* = 1315)						
Model 1	0.90	0.81, 1.00	.039	0.73	0.66, 0.81	<.001
Model 2	0.92	0.82, 1.02	.102	0.75	0.68, 0.83	<.001
Model 3	0.91	0.81, 1.01	.086	0.81	0.73, 0.91	<.001
**Tobacco** (*n* = 1081)						
Model 1	0.99	0.93, 1.05	.784	0.89	0.83, 0.95	.001
Model 2	1.00	0.94, 1.06	.894	0.90	0.84, 0.96	.001
Model 3	1.00	0.94, 1.07	.957	0.94	0.87, 1.01	.077
**Cannabis** (*n* = 1346)						
Model 1	0.98	0.94, 1.02	.287	0.90	0.86, 0.96	<.001
Model 2	0.99	0.95, 1.03	.591	0.92	0.87, 0.97	.002
Model 3	0.99	0.95, 1.04	.77	0.95	0.91, 1.00	.063

Odds ratios (OR) and 95% confidence intervals (CI) are shown

Model 1: univariate

Model 2: adjusted for sex and age (continuous)

Model 3: adjusted for sex, age, marital status, region, education, employment, housing tenure, other lifetime drug use, current use of alcohol or tobacco and year of data collection

### Relationships between age at first substance use and frequency of cannabis use among subjects who had used cannabis in the last month

Linear regression analyses exhibiting associations between age at first substance use and cannabis use frequency are presented in Table 3. Among all subjects who had used cannabis in the last month, age at first use of tobacco (β = − 0.547; *P* < .001) and cannabis (β = − 0.634; *P* < .001) were found to be independently inversely associated with greater frequency of cannabis use within the last 30 days in fully adjusted models. For younger adults aged 15–34 years, age at first use of alcohol, tobacco and cannabis were each found to be significantly inversely associated with frequency of cannabis use in the last month, with relationships for age of tobacco use (β = − 1.138; *P* < .001) and cannabis use (β = − 1.343; *P* < .001) onset being noticeably stronger than for alcohol (β = − 0.661; *P* = .023).

**Table 3:** Associations between age at first substance use and cannabis use frequency among subjects who reported using cannabis in the last month – linear regression (*n* = 394)

Age at first use	Cannabis use frequency			
	β	S.E.	95% CI	***P***-value
***All subjects***				
**Alcohol** (*n* = 386)				
Model 1	−0.807	0.25	−1.295, −0.318	.001
Model 2	−0.781	0.25	−1.269, −0.294	.002
Model 3	−0.452	0.24	−0.928, 0.023	.062
**Tobacco** (*n* = 368)				
Model 1	−0.653	0.15	−0.946, −0.360	<.001
Model 2	−0.674	0.15	−0.968, −0.379	<.001
Model 3	−0.547	0.15	−0.836, −0.258	<.001
**Cannabis** (*n* = 390)				
Model 1	−0.675	0.12	−0.918, −0.432	<.001
Model 2	−0.793	0.13	−1.042, −0.543	<.001
Model 3	−0.634	0.12	−0.878, −0.390	<.001
***Subjects aged 15–34 years***				
**Alcohol** (*n* = 298)				
Model 1	−0.962	0.29	−1.531, −0.393	.001
Model 2	−0.982	0.29	−1.552, −0.412	.001
Model 3	−0.661	0.29	−1.228, −0.093	.023
**Tobacco** (*n* = 282)				
Model 1	−1.430	0.20	−1.829, −1.032	<.001
Model 2	−1.458	0.21	−1.862, −1.054	<.001
Model 3	−1.138	0.21	−1.560, −0.717	<.001
**Cannabis** (*n* = 302)				
Model 1	−1.413	0.20	−1.801, −1.026	<.001
Model 2	−1.521	0.20	−1.912, −1.130	<.001
Model 3	−1.343	0.21	−1.751, −0.935	<.001

Unstandardized β coefficients, standard errors (S.E.) and 95% confidence intervals (CI) are shown

Model 1: univariate

Model 2: adjusted for sex and age (continuous)

Model 3: adjusted for sex, age, marital status, region, education, employment, housing tenure, other lifetime drug use, current use of alcohol or tobacco and year of data collection

### Relationships between age at first substance use and CUD

Table 4 shows associations between age at first substance use and CUD among respondents who had used cannabis in the last year. Among all subjects in this cohort, no associations were observed between age at first use of alcohol, tobacco or cannabis and CUD in full models. However, the odds of having a CUD were found to be reduced by 14% (OR = 0.86; 95% CI: 0.78, 0.94) and 11% (OR = 0.89; 95% CI: 0.82, 0.98) for each year of delayed tobacco and cannabis use onset respectively in analyses which examined younger survey participants.

**Table 4:** Associations between age at first substance use and having cannabis use disorder^a^ among subjects who reported using cannabis in the last year – binary logistic regression (*n* = 764)

Age at first use	Mean age at first use		Model 1		Model 2		Model 3	
	Cannabis use disorder	No cannabis use disorder	OR (95% CI)	***P***-value	OR (95% CI)	***P***-value	OR (95% CI)	***P***-value
***All subjects***								
Alcohol (*n* = 750)	15.15 ± 1.7	15.50 ± 1.8	0.89 (0.82, 0.98)	.012	0.89 (0.81, 0.98)	.018	0.95 (0.86, 1.06)	.354
Tobacco (*n* = 663)	14.75 ± 2.7	15.45 ± 3.2	0.92 (0.86, 0.97)	.004	0.89 (0.83, 0.95)	<.001	0.94 (0.87, 1.01)	.095
Cannabis (*n* = 758)	16.84 ± 3.4	18.10 ± 3.9	0.90 (0.85, 0.94)	<.001	0.91 (0.87, 0.96)	.001	0.96 (0.90, 1.02)	.14
***Subjects aged 15–34 years***								
Alcohol (*n* = 579)	15.22 ± 1.7	15.44 ± 1.7	0.92 (0.83, 1.02)	.112	0.92 (0.83, 1.03)	.142	0.99 (0.87, 1.12)	.867
Tobacco (*n* = 502)	14.65 ± 2.5	15.64 ± 2.4	0.84 (0.78, 0.91)	<.001	0.82 (0.76, 0.89)	<.001	0.86 (0.78, 0.94)	.001
Cannabis (*n* = 584)	16.52 ± 2.4	17.52 ± 2.4	0.84 (0.78, 0.90)	<.001	0.84 (0.78, 0.91)	<.001	0.89 (0.82, 0.98)	.012

Odds ratios (OR) and 95% confidence intervals (CI) are shown

Model 1: univariate

Model 2: adjusted for sex and age (continuous)

Model 3: adjusted for sex, age, marital status, region, education, employment, housing tenure, other lifetime drug use, current use of alcohol or tobacco and year of data collection

^a^Any cannabis abuse or dependence

## Discussion

In this study we used data from Ireland’s 2010/11 and 2014/15 National Drug Prevalence Surveys to determine relationships between age at first use of alcohol, tobacco and cannabis and patterns of cannabis use, frequency of use within the last 30 days and whether age at first substance use was related to having a CUD. For the full sample, when compared to former users, the odds of being a current cannabis user were found to be reduced by 11% and 4% for each year of delayed alcohol and cannabis use onset, respectively. Among subjects who indicated current cannabis use, significant inverse linear relationships were noted, with increasing age of first use of tobacco and cannabis being associated with a decreased frequency of cannabis use within the last 30 days. In addition, fully adjusted models demonstrated relationships between age at first use of tobacco and cannabis and having a CUD among subjects aged 15–34 years of age.

Although previous studies on the relationship between alcohol and cannabis are conflicting, our finding that earlier onset of tobacco and cannabis was associated with heavier current cannabis use has been observed in the literature [9, 12, 28, 29]. We also noted that mean age of first use of alcohol and tobacco was lower than cannabis among survey participants. Consequently, these findings could be interpreted as a supporting the sequential initiation pattern of alcohol and tobacco preceding cannabis use. However, although substance use in Western societies normally begins with alcohol or tobacco use, studies have suggested that many other factors also influence patterns of cannabis use, thus limiting the predictive ability of age of onset as a risk factor for later use of the drug [30]. These factors include context of first use, type of use, timing of use, family substance use, parental education, externalising behaviours and conduct problems [31–33].

Nevertheless, the linear associations observed in this study showing that earlier use of tobacco and cannabis was related to heavier cannabis use among current users are important in light of research demonstrating that a dose-response relationship may exist between frequency of cannabis use and adverse health effects [34], including mental health disorders, acute psychotic symptoms, abnormal cognitive development, chronic lung disease and cardiovascular disease [1, 35]. Research has also suggested that earlier onset substance use may lead to assimilation into deviant and substance-using peer groups, which in turn may lead to increased risk of escalation of substance use [36]. Proposals to liberalise cannabis laws are currently being explored in many countries, with some jurisdictions having commenced legalisation of cannabis [37]. In 2019, legislation was passed to allow for a Medical Cannabis Access Programme to come into operation in Ireland on a pilot basis for 5 years. While there has been concern that legalising cannabis for medical or adult use may increase adolescent use [38, 39], the early evidence on this is certainly not conclusive [40]. Given our finding that earlier onset use was associated with increased likelihood of progressing to a CUD, the impact of policy changes upon age of cannabis initiation will need to be monitored.

Our results showing that age of cannabis use onset was independently associated with having a CUD is also supported by the literature. In a cross-sectional sample of 8068 participants, Le Strat et al. [41] found that the younger respondents were when they began using cannabis, the greater their likelihood of experiencing a CUD. In a representative sample of 1520 youth aged 14.9–17.4 years, Swift et al. [42] found early use onset of cannabis to be related to the risk of becoming cannabis dependent. Interestingly, we additionally found earlier onset of tobacco use to be associated with having a CUD. This finding contrasts with previous research; in a prospective-longitudinal community study of 3021 subjects, Behrendt et al. [20] observed no direct cross-substance momentum of younger age at first tobacco use for the risk of developing a CUD.

It should be noted, however, that relationships between tobacco and cannabis use onset and CUD were only observed among younger survey respondents in our sample. Nevertheless, a majority (85.0%) of subjects in our study who indicated having a CUD were aged between 15 and 34 years and mean age at first use of tobacco and cannabis was lower among younger subjects compared to participants aged 35 years or older. This may partly account for observed findings. However, we also noted that relationships between tobacco and cannabis use onset and frequency of cannabis use were noticeably strong among subjects aged 15–34 years of age. Heavier cannabis use is a known risk factor for having a CUD [43]. Consequently, earlier tobacco and cannabis use may be a contributor to the more intense cannabis use necessary for CUD development [20] and this may be of particular importance among younger adults.

In Ireland, the type of cannabis being used has changed in recent years, from relatively low potency resin (‘hash’) to higher potency herbal cannabis (‘weed’) [7, 44]. This trend in use of increasingly potent herbal cannabis has also occurred across Europe [45]. High-potency cannabis is associated with an increased severity of dependence, especially in young people [46]. The increase in use and move towards higher potency products in Ireland has coincided with a substantial increase in treatment presentations [7]. Drug treatment services have traditionally been funded in the absence of comprehensive, quantitative planning models [47]. However, planning models based on the needs of the population are important for the successful implementation of treatment services and to adequately plan these services requires an understanding of the population in need of treatment [48]. From a secondary prevention perspective, it is also necessary to know who is at greatest risk of progressing from substance initiation to riskier patterns of future use. Finding from this research suggest that in Ireland, younger adult cannabis users who report a more precocious pattern of early use of substances, including alcohol and especially tobacco and cannabis, should be targeted.

### Strengths and limitations

This research has several strengths. This is the first study to examine relationships between age of alcohol, tobacco and cannabis use onset and cannabis use patterns, frequency of cannabis use and CUD, using a sample of lifetime cannabis users from Ireland. A further strength of this research is the large sample size which utilised data from two population studies that used the same field survey procedures and data collection methods and which were weighted and adjusted for the year of data collection; thus, our findings are generalisable to the whole population. We also controlled for important potential confounders in analyses and used valid and reliable measures of cannabis abuse and dependence, defined using the DSM-IV, and Ireland is one of the first countries in Europe to include these variables in two general population surveys. Research on relationships between substance use onset and cannabis use is important for preventative work and for informing and assessing drug policy. This is especially relevant at the present time given the debate regarding the decriminalisation of cannabis in many countries.

Despite these strengths, several limitations should be noted. The cross-sectional study design limits inference with regard to causality and precludes drawing conclusions regarding the temporal direction of relationships. Nevertheless, while a cross-sectional study has reduced ability to identify direction of causality in relationships, it has the advantage of including a much wider age range than typical prospective studies. Another limitation of this research is the use of self-reported questionnaires which are subject to potential inaccuracies, recall and reporting bias. Therefore, residual confounding arising from imprecise measurement of variables should also be considered. Also, we did not have data on other substance use disorders or psychiatric disorders for either survey and these may be important potential confounders. In addition, due to sample size constraints, we analysed cannabis abuse and dependence together rather than as separate constructs. Research examining abuse and dependence separately may be warranted as abuse or dependence criteria may be differentially predictive of adverse outcomes.

## Conclusions

In conclusion, the results from this study of a sample of lifetime cannabis users demonstrate an association between age of alcohol and cannabis use onset and current cannabis use. Subjects who had used tobacco and cannabis at an earlier age were more likely to be heavier users of cannabis. Age of onset of tobacco and cannabis use was significantly related to having a CUD among younger survey respondents. Consequently, these findings suggest that among people who report past cannabis use, it is those with a more precocious pattern of early use of substances, including alcohol and especially tobacco and cannabis, who are more likely to report ongoing, heavy and problematic cannabis use. Prevention initiatives should prioritise people with a pattern of very early onset tobacco or cannabis use. Findings from this study may be used to better inform public health efforts to improve secondary prevention of problematic drug use in the Irish and other populations.

## Data Availability

The data used and analysed for the purpose of this study are available from the corresponding author on reasonable request.

## Abbreviations

CI: Confidence Interval
CUD: Cannabis Use Disorder
DSM: Diagnostic and Statistical Manual of Psychiatric Disorders
EMCDDA: European Monitoring Centre for Drugs and Drug Addiction
GDPR: General Data Protection Regulation
M-CIDI: Munich Composite International Diagnostic Interview
OR: Odds Ratio
